# Association of periodontitis and tooth loss with cognitive decline in the older adults - a systematic review

**DOI:** 10.1186/s12903-026-08310-w

**Published:** 2026-04-11

**Authors:** Afsary Jahan Khan, Iffat Rahman, Hasina Akhtar Beg, Shahinur Akter, Rashida Haque, Dil Tahmina Monsur, Haslina Rani, Tuti Ningseh Mohd Dom

**Affiliations:** 1 Department of Family Oral Health, Faculty of Dentistry, Universiti Kebangsaan Malaysia, Jalan Raja Muda Abdul Aziz, Kuala Lumpur, 50300 Malaysia; 2 Institute of Biological Sciences, University of Rajshahi, Rajshahi, 6025 Bangladesh; 3 School of Business & Management, Lincoln University College (LUC), Jalan Stadium, Petaling Jaya, 47301 Malaysia; 4 Faculty of Preventive and Social Medicine, Bangladesh Medical University (BMU), Dhaka, Shahbag 1000 Bangladesh; 5 University of Dhaka, Dhaka, Bangladesh; 6 The School of Nursing, Murdoch University, Murdoch, Perth, Western Australia 6150 Australia

**Keywords:** Older adults, Periodontitis, Tooth loss, Dementia, Cognitive impairment, Alzheimer’s disease.

## Abstract

**Background:**

The increasing global burden of dementia highlights the importance of identifying factors that may contribute to cognitive decline in later life. Growing evidence suggests that chronic oral conditions, particularly periodontitis (PD) and tooth loss, may be associated with Alzheimer’s disease (AD) and related dementias. This study synthesizes current observational evidence on the association between PD, tooth loss, and cognitive impairment (CI) among older adults.

**Methods:**

A comprehensive literature search was conducted in PubMed, the Cochrane Library, Embase, Scopus, and Google Scholar for English-language studies published between 2010 and 2025. Cross-sectional and longitudinal cohort studies examining associations between PD, tooth loss, and CI were included. Study selection, data extraction, and quality assessment were performed in accordance with PRISMA 2020 guidelines.

**Results:**

Thirteen studies met the inclusion criteria, with sample sizes ranging from 40 participants to over 500,000 individuals in large population-based cohorts. Most studies focused on adults aged ≥ 50 years, particularly those aged 60 years and above. Periodontal status, tooth loss, and cognitive outcomes were assessed using heterogeneous diagnostic methods. Most studies reported significant associations between PD or tooth loss and CI, dementia, or AD. Periodontal treatment appeared protective in several studies, although some associations weakened after adjustment for confounders.

**Conclusions:**

The findings support PD and tooth loss are consistently associated with adverse cognitive outcomes, although causal relationships cannot be established due to methodological heterogeneity and residual confounding. Integrating oral health care into geriatric and dementia-prevention strategies may help preserve cognitive function and improve quality of life among older adults.

**Supplementary Information:**

The online version contains supplementary material available at 10.1186/s12903-026-08310-w.

## Introduction

Cognitive disorders, including dementia and Alzheimer’s disease (AD), are progressive neurodegenerative conditions that impair memory, reasoning, language, and daily functioning [1]. Globally, an estimated 51.6 million people, approximately 0.7% of the population are living with dementia, with prevalence more than doubling between 1990 and 2019 [2]. Despite this growing burden, awareness of dementia remains limited, particularly among older adults who are at increased risk of developing mild cognitive impairment (MCI) [3]. These trends underscore the urgent need to identify factors that may contribute to cognitive decline and inform strategies to promote healthy cognitive ageing.

Oral health (OH) has emerged as a promising area of interest in this regard. Periodontitis (PD), a chronic inflammatory disease of the tooth-supporting tissues, and tooth loss, its ultimate consequence, have been increasingly linked to cognitive decline [4, 5]. For the purpose of this systematic review (SR), ‘periodontitis’ refers specifically to the chronic inflammatory condition, while ‘periodontal disease’ allows for broader definitions used in some studies [6]. ‘Cognitive decline’ is used here as an umbrella term encompassing mild cognitive impairment (MCI), dementia, and Alzheimer’s disease (AD) [7]. In addition, tooth loss may serve as an indicator of previous severe periodontal infection, periodontitis can contribute to tooth loss, but missing teeth also result from caries, trauma, or dental extractions unrelated to infection [8]. A systematic review has demonstrated that individuals with a higher number of missing teeth often had advanced periodontitis [9]. Epidemiological studies show that individuals with long-term exposure to periodontal pathogens have a higher risk of developing AD [10], while tooth loss is independently associated with poorer memory and greater risk of dementia [11, 12]. In addition, tooth loss and periodontitis, both resulting from oral bacterial infections, have recently been proposed as potential risk factors for AD and related dementias [13, 14]. Given their high global prevalence [15], assessing their possible role in the onset and progression of dementia is of significant importance.

Interpretation of these associations, however, requires conceptual caution. As the majority existing evidence is observational, the associations between periodontitis, tooth loss and cognitive outcomes may reflect shared determinants such as age, education, comorbidity and health behaviors, as well as potential reverse causation [16]. This SR therefore synthesize the evidence with explicit attention to measurement heterogeneity and confounding, to support accurate interpretation and identify priorities for future longitudinal research.

This interpretation aligns with broader geriatric perspectives that conceptualize age-related declines in oral function including tooth loss, reduced chewing ability, impaired swallowing, and compromised oral hygiene, as indicators of cumulative functional decline rather than isolated disease entities [17, 18]. While several studies have reported associations between reduced dentition, impaired mastication, and adverse cognitive outcomes [17, 19]. This lifespan perspective suggests that late-life OH may serve as a proxy for lifelong risk factors, rather than a direct cause of cognitive decline [16].

A bidirectional relationship between OH and cognitive impairment (CI) has also been proposed, whereby CI may exacerbate poor OH through reduced self-care capacity and limited access to dental services [18, 20]. However, the present SR focuses specifically on PD and tooth loss in relation to cognitive outcomes to allow a more focused and critical appraisal of how these oral conditions have been examined and interpreted within cognitive ageing research. While several oral function parameters (e.g. chewing ability, saliva flow) have been linked to cognition, however, this SR focuses on tooth loss because it is the most consistently reported functional marker across population-based studies and is frequently analyzed alongside periodontitis [16].

Prior reviews have often examined periodontal conditions or tooth loss in isolation, or have focused on narrower cognitive outcomes. Given the rapid growth and heterogeneity of the observational literature, an updated synthesis addressing both exposures is needed [21]. This SR synthesizes observational evidence on associations between PD and tooth loss and CI outcomes (including MCI, dementia and AD) in older adults, and summarizes key sources of heterogeneity that influence interpretation.

## Materials and methods

### Protocol development and registration

This SR employed a structured literature review methodology to comprehensively examine association between periodontitis, tooth loss, and cognitive decline among the older adults.

The SR was conducted following the Joanna Briggs Institute (JBI) methodology for systematic reviews of effectiveness and reported in accordance with the PRISMA guidelines [22]. The protocol was registered with the International Prospective Register of Systematic Reviews (PROSPERO) [23] under the Registration ID: CRD420251168698. A summary of the SR process is provided in Table 1.

**Table 1:** Summary of search strategy

Phase 1	Research Questions
Formulation of Research questions	Primary Research Question: Q1. What is the association between PD, tooth loss, and cognitive decline among the older adults? Sub-Questions: Q1. What diagnostic approaches have been used to assess PD, tooth loss, and cognitive outcomes among older adults in existing studies? Q2. To what extent are periodontitis and tooth loss associated with an increased risk of cognitive decline, dementia, and Alzheimer’s disease in older adults? Q3. What confounding factors and comorbid conditions influence the association? Q4. What gaps and methodological limitations exist in the current literature examining the relationship between periodontitis, tooth loss, and cognitive disorders among older adults?
Phase 2	
Inclusion criteria (PICOS)	Population Intervention Comparison Outcomes Study design
Exclusion criteria	
Phase 3 Selection of databases,	Electronic databases- PubMed (https://pubmed.ncbi.nlm.nih.gov) Cochrane Library (https://www.cochranelibrary.com) Embase (https://www.embase.com) Google Scholar (https://scholar.google.com) Scopus (https://www.scopus.com)
Search settings, and	Database setting: Journal articles Language setting: English language only Search time setting: 1st March 2010 to 1st March 2025
keywords	MeSH Terms (Table 2) Emtree Terms
Phase 4 Search debugging and refinement	Article Selection PRISMA • Identification • Screening • Eligibility checking • Final selection
Phase 5 Data Coding, Data Assessment, Quality Appraisal, and Data Synthesis	Data Extraction Data Analysis Qualitative Analysis Quality Assessment/ Risk of Bias Assessment of Included Studies

The review process was structured into five key phases:

1. Formulation of research questions.
2. Inclusion and exclusion criteria.
3. Selection of databases, search settings, and keywords.
4. Search debugging and refinement.
5. Data coding, data assessment, quality appraisal, and data synthesis.

Each phase is detailed in the sections below.

#### Phase 1: Formulation of research questions

The primary aim of this study was to systematically explore the association between PD, tooth loss, and cognitive decline among the older individuals.

To guide the review, research questions were developed and are presented in Table 1. Accordingly, the primary aim of this study was to systematically synthesize current evidence on the association between PD, tooth loss, and cognitive decline among older adults.

The other objectives were:

1. To evaluate the diagnostic approaches used to assess PD, tooth loss, and cognitive outcomes, and to determine the extent to which these oral conditions contribute to the risk of developing dementia and AD;
2. To identify potential confounding factors and comorbid conditions that may influence the association between PD, tooth loss, and cognitive decline; and.
3. To identify the current gaps and limitations in the literature on the relationship between PD, tooth loss, and cognitive disorders in older adults, and to propose directions for future research and public health policy.

#### Phase 2: Inclusion and exclusion criteria

Inclusion and exclusion criteria were defined prior to the literature search. This SR specifically focused on studies that examined PD and tooth loss, and CI outcomes such as cognitive decline, MCI, dementia or AD. Studies were eligible if both PD and tooth loss were assessed within the same study population, even if one exposure was treated as the primary variable and the other as a secondary measure or proxy indicator. In studies using administrative databases, variables such as tooth extraction history or treatment codes were considered acceptable indicators of advanced periodontal disease when interpreted by the original authors within a periodontal framework. This approach was adopted to capture the range of operational definitions used in epidemiological research while maintaining conceptual consistency.

##### Inclusion criteria

Studies were included if they were published in peer-reviewed journals, and were in English. To choose the relevant literatures, the following PICOS criteria were followed:

- Population (P): Older adults (with age cutoff as defined by each study; typically, ≥ 50 or 60 years) assessed for PD, tooth loss, and cognitive status. Adults described as older in included studies (commonly ≥ 60 years; some cohorts included ≥ 50 years to enable long-term follow-up into older age).
- Intervention (Exposure): Periodontitis/periodontal disease and tooth loss (number of missing teeth, edentulism or extraction history, as measured).
- Comparison (C):
  Older adults without PD and/or without significant tooth loss,
  In some studies, types of severities of PD/tooth loss were compared.
- Outcomes (O): Risk of cognitive decline, including mild cognitive impairment (MCI) and dementia or AD or other types.
- Settings/Study designs (S): Observational studies such as cohort, case-control, and cross-sectional that assessed the association between PD, tooth loss, and cognitive decline.

Studies were included if they assessed both periodontitis and tooth loss, regardless of whether one was the primary exposure and the other a covariate. Studies using proxy indicators (e.g., tooth extraction history as a surrogate for periodontitis) were also included but noted for methodological limitations. We used an inclusive age approach because international sources commonly use 60+ (with 65 also common), and several epidemiological studies enroll late midlife adults to capture cognitive outcomes after long follow-up. 

##### Exclusion criteria

The following studies were excluded from this study:

- Adults below 50 years old were excluded. However, longitudinal studies with baseline participants younger than 50 were included provided they reported cognitive outcomes for older adults (≥ 50 years) at follow-up.
- Studies that addressed only one component, either PD or tooth loss - were excluded.
- Non-empirical studies, including editorials, opinion pieces, and narrative reviews.
- Review articles of any kind (e.g., systematic reviews, scoping reviews).
- Abstract-only publications, such as conference proceedings without full-text articles.
- Articles not published in English, as the research team lacked the linguistic expertise to ensure accurate translation, and machine translation tools such as Google Translate may introduce inaccuracies.
- Studies published before March 2010, to ensure the inclusion of only recent and relevant papers.
- Gray literature, including unpublished theses, reports, and preprints.

#### Phase 3: Database selection, settings, and keywords

Two experienced researchers independently conducted comprehensive searches across multiple electronic databases, including PubMed (via MEDLINE), the Cochrane Library, Embase, Google Scholar, and Scopus (summarized in Table 1). The initial search strategy was developed and then adapted to suit the syntax and controlled vocabulary (e.g., MeSH terms in PubMed, Emtree terms for Embase) specific to each database. Search Terms are mentioned in Table 2.

**Table 2:** Keywords and databases search strategy

Databases	Query	Notes	Records retrieved	Filter
PubMed (via MEDLINE)	(“periodontal disease” OR “periodontitis” OR “gum disease” OR “gingivitis” OR “tooth loss” OR “missing teeth” OR “edentulism” OR “edentulous” OR “tooth extraction”) AND (“cognitive decline” OR “cognitive impairment” OR “dementia” OR “Alzheimer disease” OR “neurocognitive disorder”) AND (“elder persons” OR “older adult” OR “older people” OR “geriatric” OR “senior” OR “healthy aging”)	Used MeSH + keywords; balanced specificity and sensitivity.	419	*Publication date: March 2010 to March 2025 *Document type: Article *Language: English
Cochrane Library	(“periodontal disease” OR “periodontitis” OR “gum disease” OR “gingivitis” OR “tooth loss” OR “missing teeth” OR “edentulism” OR “edentulous” OR “tooth extraction”) AND (“cognitive decline” OR “cognitive impairment” OR “dementia” OR “Alzheimer disease” OR “neurocognitive disorder”) AND (“elder persons” OR “older adult” OR “older people” OR “geriatric” OR “senior” OR “healthy aging”)	Similar to PubMed but adapted to Cochrane’s search interface.	27	
Embase	(“periodontal disease” OR “periodontitis” OR “gum disease” OR “gingivitis” OR “tooth loss” OR “missing teeth” OR “edentulism” OR “edentulous” OR “tooth extraction”) AND (“cognitive defect” OR “cognitive impairment” OR “cognitive decline” OR “dementia” OR “Alzheimer disease” OR “neurocognitive disorder”) AND (“aged” OR “elder” OR “older adult” OR “older people” OR “geriatric” OR “senior” OR “healthy aging”)	Includes Emtree terms to maximize coverage of indexed terms.	9	
Google Scholar	(“periodontitis” OR “periodontal disease” OR “tooth loss” OR edentulism OR edentulous OR “tooth extraction”) AND (“dementia” OR “cognitive decline” OR “cognitive impairment” OR “mild cognitive impairment” OR “Alzheimer disease” OR “Alzheimer’s disease”) AND (“older adult” OR “older person” OR elderly OR geriatric OR senior OR “healthy aging”)	GS does not support advanced Boolean nesting; kept broad but structured.	252	
Scopus	TITLE-ABS-KEY (“periodontal disease” OR “periodontitis” OR “gum disease” OR “gingivitis” OR “tooth loss” OR “missing teeth” OR “edentulism” OR “edentulous” OR “tooth extraction”) AND TITLE-ABS-KEY (“cognitive decline” OR “cognitive impairment” OR “dementia” OR “Alzheimer disease” OR “neurocognitive disorder”) AND TITLE-ABS-KEY (“elder” OR “older adult” OR “older people” OR “geriatric” OR “senior” OR “healthy aging)	Restricted to TITLE-ABS-KEY for precision and relevance.	183	
	**Total**	**890**		

The search had no geographical or publication type restrictions, however, it was limited to articles published in English between March 2010 and March 2025, ensuring a focus on recent and relevant advancements in the field. The search strategy was designed using the PICOS framework, incorporating Medical Subject Headings (MeSH) and Emtree terms, keywords, Boolean operators (AND, OR), and phrase searching to optimize sensitivity and specificity (Table 2) for keyword combinations).

An initial pairwise keyword search was conducted in March 2025, using one term from each PICOS category. The same search strategy and syntax were consistently applied across all databases, with a specific focus on studies related to association between PD, tooth loss, and cognitive decline among older adults. A follow-up search in August 2025 was carried out to capture any newly published studies. Detailed search strings and database-specific strategies are provided in the supplementary material (Table 2).

To enhance the thoroughness of the search, a manual search of reference lists from relevant studies and previously published SR was also performed to identify additional eligible articles.

#### Phase 4: Search debugging

Records were de-duplicated and screened in two stages (title/abstract, then full text) by two reviewers independently. Discrepancies were resolved through discussion and, if required, consultation with a third reviewer. Reasons for full-text exclusions were documented and are summarized in the PRISMA flow diagram.

#### Phase 5: Data coding and data assessment

A standardized data extraction form was developed, pilot-tested on three studies, and refined to ensure comprehensive and accurate capture of relevant data. To promote consistency across reviewers, a calibration exercise was conducted prior to the full extraction process.

Data extraction was performed by the same authors using the finalized data collection form. All extracted information was systematically documented in an Excel spreadsheet to capture key study characteristics. Inter-reviewer disagreements were minimal and were resolved through discussion, with consultation from a third reviewer when necessary.

The extracted data from each article included:

1. Bibliographic: author’s name, year of publication, and country/regions.
2. Study characteristics: study setting, study design, sample size, follow-up duration.
3. Population: age (mean/median, range), sex distribution, confounding factors and comorbidities.
4. Exposure: PD (stages/grades), tooth loss (edentulism, number missing, categories (0–8, 9–20, > 20), CI (different types of tests).
5. Conclusions/ Outcomes.

### Assessment of study quality (Methodological)

The methodological quality of the included studies was assessed using the Joanna Briggs Institute (JBI) Critical Appraisal Checklist, which is designed for evaluating all the three types of studies: cohort, cross-sectional and case-control studies.

This SR adopted the JBI tool, which comprises two sections with a total of 12 core questions (refer to Tables 5 and 6). Two researchers independently appraised each study’s quality. Any disagreements were resolved through discussion or, when necessary, by consulting other researchers.

The risk of bias for each study is summarized in Tables 5 and 6, identified methodological limitations that may influence the interpretation of results. Notably, all included studies proceeded to data extraction and synthesis regardless of their quality scores, ensuring a comprehensive and inclusive review process.

### Data synthesis and thematic analysis

A thematic content analysis was conducted to identify recurring patterns and concepts across the included studies. Due to substantial clinical, methodological, and statistical heterogeneity, meta-analysis was not feasible. Each study was systematically reviewed and coded for key characteristics, including study design and setting, diagnostic criteria for PD, tooth loss, and CI, as well as reported confounding factors and comorbidities. These elements were synthesized to enable structured comparison of findings and to examine how PD and tooth loss related to cognitive outcomes. In addition to summarizing existing evidence, this approach highlighted important knowledge gaps and methodological limitations, thereby informing priorities for future research and strengthening the relevance of the findings for clinical practice and public health.

## Results

### Search results of the studies

Thirteen articles between March 2010 and March 2025 were included in this SR for descriptive and categorical analyses. The article selection process in this study followed the four key steps of the PRISMA framework: identification, screening, eligibility, and inclusion. Every step is described below:

#### Identification

An initial database search yielded 890 articles. An additional 9 relevant studies were identified through manual searches from previously published reviews. Thus, initially a total of 899 articles were initially identified for consideration.

#### Screening

Duplicate records were removed using EndNote X9, reducing the number of articles from databases search 890 to 796. No duplicate recorded for manual search. Two reviewers independently screened titles and abstracts for relevance. Based on the inclusion criteria, 577 articles were excluded due to irrelevance, leaving 364 articles for full-text review. Additionally, a screening of the reference lists of these 364 articles led to the identification of more potentially relevant 12 studies.

#### Eligibility

**Fig. 1:**
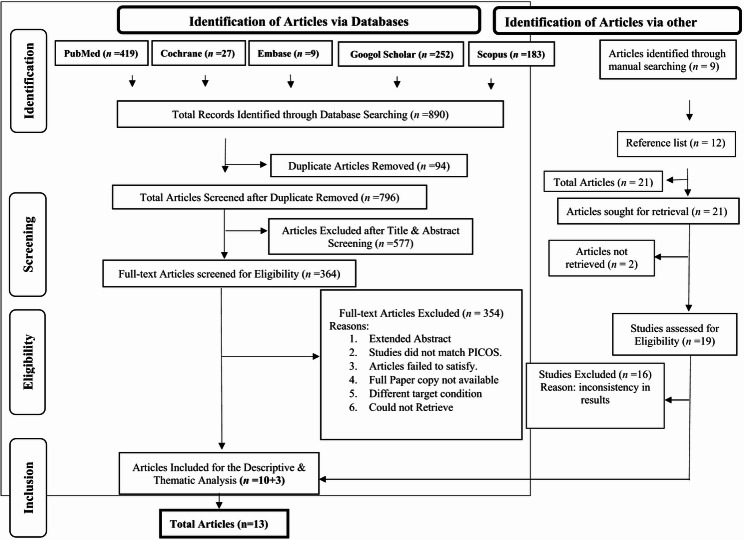
PRISMA Flowchart

The full texts of all 385 articles (databases, manual search and reference lists) were read and assessed in detail. Disagreements during the eligibility assessment were resolved through discussion, and when necessary, with input from the third and fourth reviewers. Where multiple publications from the same study were found, they were linked and considered as a single unit. Every reason for exclusion at the full-text review stage was documented systematically (Fig. 1).

#### Final selection

Finally, a total of 13 articles (10 from databases and 3 from manual search & reference lists) met all eligibility criteria and included for the descriptive and thematic analysis. These studies formed the basis for the subsequent analysis and synthesis of findings related to the association between PD, tooth loss, and cognitive decline among the older persons.

The complete article selection process, including inclusion and exclusion flow, is illustrated in the PRISMA flow diagram (Fig. 1).

### Source country

As shown in Table 3, the USA contributed the highest number of publications, with four studies [25, 29, 31, 32] and then Japan with three studies [28, 33, 35]. The remaining countries were represented by a single study each: Brunei [24], Korea [26], Germany [27], Sweden [30], Denmark [14], and Taiwan [34].

### Study settings and study designs

Study settings varied from community-dwelling populations (e.g., Japan, Sweden, USA) to hospital-based cohorts (Germany, Denmark), and large-scale national insurance/health database analyses (Taiwan, Korea, Brunei, USA).

Of the thirteen studies (*n* = 13), all were journal articles comprised of prospective cohort studies [25, 29, 31, 28], retrospective cohorts [24, 34], cross-sectional analyses [14, 27, 30, 32], and a few longitudinal observational designs [35, 33, 26] (Table 3).

**Table 3:** Characteristics of the included studies

No	Author, Year & Country	Settings	Study Design Sample Size & Age Group	Follow-up Duration	Confounding Factors (CFs) & Comorbidities (CMs)	Conclusions	Limitations
1	Fadzli et al. (2024) [24] Brunei	Individuals admitted to government healthcare settings. (Using Brunei Health Information Management System (Bru-HIMS) data, from 2014 to 2023).	**Study Design**: Retrospective study **Sample Size**: *n* = 76 (Female 35 Male 41) (recorded data) **Age Group**: 59 years and above (59–69, 70–79, 80+).	Up to 5 years	**CFs**: Age, gender, ethnicity. **CMs**: Diabetes, hypertension, hyperlipidemia exacerbate AD progression in periodontitis patients.	1. A strong association between PD and AD, revealed that common dental treatments such as tooth extraction may increase AD risk due to inflammation and physiological stress. 2. Addressing chronic periodontitis severity specifically may help reduce risks in affected populations. 3. Women with gingivitis/chronic PD may be more vulnerable to AD. 4. Periodontal therapy may help mitigate dementia risk.	1. Reliance on medical records → risk of incomplete data and selection bias. 2. Small sample size (*n* = 76). 3. Lack of prospective follow-up to establish causality. 4. Biological mechanisms (e.g., inflammatory markers, bacterial detection in brain tissue) not directly measured. 5. ⁠Confounding factors like socioeconomic status, education, and lifestyles were not fully surveyed.
2	Kaye et al. (2010) [25] United States	Community dwellers (Veterans Affairs Dental Longitudinal Study data).	Study design: Prospective study. Sample size: *n* = 597 All Male (recorded data) Age Group: 28 to 84 years. Adults ≥ 50 years	Median follow-up of 10 years.	**CFs**: Age, Education, smoking, alcohol intake **CMs**: Diabetes mellitus, hypertension, dyslipidemia, cerebrovascular disease, ischemic heart disease, depression.	1. PD significantly increases risk of developing AD. 2. Long-term periodontal management may lower dementia risk. 3. Risk was higher in participants with multiple comorbidities.	1. Reliance on administrative data/ICD codes may misclassify diagnoses. 2. Tooth loss not clearly differentiated from other extractions. 3. Lack of clinical parameters (e.g., probing depth, attachment loss). 4. No information on oral hygiene behaviors or lifestyle factors.
3	Kim et al. (2020) [26] Korea	National Health Insurance Service, population-level health screening.	Study design: Longitudinal Retrospective Cohort Study. Sample size: *n* = 514,866 Male = 14,474; Female = 5,756 Age group: 40 to 79 years.	long-term follow-up over 14 years.	**CFs**: Age, sex, income, insurance status, BMI, total cholesterol level, smoking status, drinking status, physical activity, basic OH status **CMs**: Hypertension, diabetes mellitus, cardiovascular diseases	The risk of AD was significantly higher in patients with severe periodontitis with 1–9 remaining teeth after adjustment for sociodemographic factors, anthropomorphic measurements, lifestyle factors, and comorbidities.	1. Cause of tooth loss was not assessed; other factors beyond periodontitis (e.g., caries, trauma, orthodontics) may have contributed. 2. Dementia severity was not evaluated due to limited records. 3. Genetic and familial risk factors (e.g., APOE ε4, family history) were not considered, which may have influenced associations.
4	Laugisch et al. (2021) [27] Germany	Hospital based	Study design: Cross-sectional pilot study Sample size: *n* = 40 Female-17 Male-23 Age range: 50–70 years	Not applicable	**CFs**: Age, gender **CMs**: Excluded patients with diabetes mellitus, anemia, current smokers.	1. Both AD and other dementia patients had periodontitis with no significant differences in oral/periodontal status. 2. Patients with dementia (all forms) require special dental care to improve oral and periodontal health, particularly in institutionalized settings.	1. Small sample size (*n* = 40). 2. Cross-sectional design (no longitudinal association between PD, tooth loss, and CI progression could be determined). 3. Relatively young dementia cohort (58.3 ± 5.2 to 61.1 ± 9.9 years), limiting generalizability to older adults. 4. Exclusion of common comorbidities (e.g., diabetes, smoking) may not reflect real-world dementia populations. (this study did not mention limitations separately)
5	Yamaguchi et al. (2023) [28] Japan	Community-dwelling older adults	Study Design: Prospected Cohort Study Sample Size: *n* = 172 (gender not mentioned) Age Group: At least 55 years	13 years	**CFs**: Age sex(female), education level, smoking history, drinking history, high sensitivity C-reactive protein, BMI, number of teeth, periodontal pocket depth **CMs**: Adjusted for hypertension, diabetes mellitus, stroke, heart disease, smoking, alcohol consumption, Hypercholesterolemia	1. Fewer teeth and periodontal disease were associated with an increased risk of cognitive decline and dementia. 2. Severe tooth loss (< 10 teeth) significantly predicted cognitive impairment over 13 years. 3. Findings support the role of oral frailty (tooth loss + PD) in the risk of later cognitive decline.	1. Self-reported health behaviors (possible recall bias). 2. Lack of detailed clinical periodontal measurements beyond probing depth. 3. Residual confounding could not be ruled out. 4. Study conducted in one geographic region (Japan), which may limit generalizability.
6	Luo et al. (2023) [29] USA	Community-based clinical examination centers in four U.S. metropolitan areas.	Study Design: Prospective cohort Study. Sample size: *n* = 5709. Male = 41.9%; Female = 58.1%. Age Group: 50 to 74 years.	Oral health assessed at Visit 1 (2006–2013); MCI assessed at Visit 2 (2013–2018). Models adjust for time elapsed between Visits 1 and 2 (years). (Mean interval not reported.)	**CFs**: Age, age at immigration, sex, Hispanic / Latino background, socio-economic status, health behavior, inflammatory marker, chronic condition **CMs**: cardiovascular risk factors, diabetes, general cognitive impairment.	Significant tooth loss was linked to increased risk of mild cognitive impairment, while periodontitis showed inconsistent association, highlighting the need for further research on their roles in dementia	1. Cause and timing of tooth loss unknown. 2. Denture use data unavailable. 3. Edentulous participants excluded (no total tooth loss analysis). 4. Reason for immigration unavailable. 5. Prevalence (cross-sectional) analysis of MCI, temporal order unclear; possible bidirectional relationship between OH and cognition.
7	Nilsson et al. (2018) [30] Sweden	Population-based cohort from the Swedish National Study on Aging and Care (SNAC)	Study Design: Cross-sectional study Sample size: *n* = 775 (gender not mentioned) Age Group: 60–99 years, Young old (60–66 years) Old (72–78 years) Old-old (≥ 81 years)	6-year follow-up wave.	**CFs**: Age. sex, education level **CMs**: Not mentioned	1. Bone loss and fewer teeth linked to lower MMSE scores after adjustment for age, gender and education. 2. Presence of periodontal pockets ≥ 5 mm was not significantly associated with cognitive test outcome. 3. Authors conclude that history of periodontitis (bone loss) and tooth loss may be important for cognitive function in older adults, but causality cannot be inferred from this design.	1. Cross-sectional design. 2. Panoramic radiographs used for bone-level assessment, may have distortion. 3. Edentulous participants excluded (*n* = 102) and 170 participants lacked dental exam - non-examined were older and had lower cognitive scores → possible selection bias. 4. Limited covariate adjustment in main models (only age, gender, education), residual confounding possible (e.g., vascular disease, diabetes, smoking not adjusted in presented models)
8	Naorungroj et al. (2015) [31] USA	Two U.S. communities: Forsyth County, NC; Jackson, MS.	Study Design: Prospective Cohort Study Sample size: *n* = 911 (gender not mentioned) (recorded data) Age Group: 52 to 75 years	Mean interval 7.6 ± 1.0 years (median 8 years)	**CFs**: age, race, sex, educational level, income, and study sites and education level, nutritional status, self-care abilities. **CMs**: cardiovascular risk factors, apolipoprotein E (APOE) genotype, stroke, and coronary heart disease	1. Edentulous participants had **l**ower cognitive test scores compared with dentate participants. 2. Neither edentulism, number of teeth, nor periodontal disease predicted a greater rate of cognitive decline over the 8-year follow-up. In other words, poor oral health at baseline was associated with lower cognitive performance but did not predict faster subsequent decline in this late-middle-aged sample.	1. Only three cognitive tests (two cognitive domains) were administered. The study did not use clinical dementia/ MCI diagnostic criteria. 2. Single baseline oral health assessment 3. Only two ARIC sites (Forsyth and Jackson) contributed to the longitudinal cognitive follow-up, limited generalizability. 4. The analytic sample had modest cognitive decline over follow-up (late middle-aged group), possibly limiting power to detect associations with decline.
9	Scherer et al. (2020) [32] United States	Different states of U.S. (Analysis of state level public epidemiological databases)	Study design: Cross sectional correlational study. (existing data) Sample size: Subjective Cognitive Decline (SCD): 227,393 participants. AD: 4.8 million participants. (gender not mentioned) Age: Adults ≥ 45 years for SCD prevalence. Adults ≥ 65 years for AD	Not applicable	**CFs**: No individual-level adjustment possible. **CMs**: No individual-level adjustment possible.	1. Poor oral health metrics (tooth loss, edentulism, fewer dental visits, higher PD prevalence) significantly correlated with higher AD mortality, AD prevalence, and SCD prevalence at the state level. 2. Strongest associations: SCD prevalence with lack of dental visits (*r* = − 0.69), and severe PD prevalence with SCD. 3. Findings support oral health, particularly PD and tooth loss, as potential modifiable risk factors for AD and cognitive decline	1. Ecological, state-level data, no individual-level causation can be inferred. 2. Confounding factors (comorbidities, ethnicity, education) not controlled. 3. Cross-sectional design, temporality unclear. 4. Reliance on self-reported survey data, possible reporting bias.
10	Hatta et al. (2018) [33] Eastern and Western Japan	Community Dwelling	Study design: Longitudinal study Sample Size: *n* = 463 (Male 231, Female 232) Age group: 79–81 years	3 years	**CFs**: Education level, sex, economic status, living alone or not, smoking habit, drinking habit, frequency of outing, frequency of interaction, **CMs**: Diabetes, hypertension, Dyslipidemia, stroke, malignant tumor, depression tendency, oral health status.	1. Number of teeth and mean periodontal pocket depth were not significantly associated with cognitive decline. 2. Findings suggest that maintaining posterior occlusal support may reduce the risk of cognitive decline in old-old adults, though causality is not proven. 3. A lack of posterior occlusal support at baseline predicted the incidence of cognitive decline during the subsequent 3 years in older Japanese people, even after considering for other risk factors.	1. Participants were not representative of the general older Japanese population → possible selection bias. 2. CI was defined by an arbitrary MoCA-J cut-off (≥ 3-point decrease), no standardized definition exists. 3. The study couldn’t make the detail how long participants had lacked posterior occlusal support. 4. Effect of dentures was not evaluated, most participants without posterior support wore dentures, however, cognitive decline still occurred.
11	Kamer et al. (2020) [14] Denmark	Hospital Based	Study Design: Cross-sectional Study Sample size: *n* = 152 (Female 48%, Male 52%) Age Group: 70 years	Not applicable	**CFs**: Gender, education, previous cognitive score, smoking habit, alcohol consumption **CMs**: Diabetes, hypertension, cardiovascular diseases, triglyceride level, total cholesterol level, dental status	Periodontal inflammation may adversely affect cognition in older adults, however, tooth-loss effects are partly explained by prior cognition and education.	1. Relatively small sample especially for some analyses. 2. Cross-sectional analysis at age 70 (though adjusted for prior cognition at 50), limits causal inference and directionality. 3. Limited number of cognitive tests didn’t cover all thinking aspects. 4. MCPI cannot fully distinguish gingivitis vs. periodontitis or capture cumulative inflammatory burden. 5. Analytic sample had higher education and cognition than excluded subjects; homogeneous Danish cohort limits external generalizability.
12	Lee et al. (2017) [34] Taiwan	Taiwan National Health Insurance Research Database	Study design: Retrospective Cohort Study Sample size: *n* = 182,747 (Female 92,296 Male 90,451) (recorded data) Age group: 45 to 75 years	10 years maximum	**CFs**: Age, sex, monthly income, residential urbanicity, and pre-existing comorbidities **CMs**: Hypertension, Diabetes mellitus, Hyperlipidemia, genetic factor (APOE E)	1. Individuals with severe periodontal disease or no PD treatment had a higher risk of developing dementia. 2. Those who received dental prophylaxis or intensive PD treatment had significantly lower dementia risk. 3. Suggests that treating periodontal disease may help reduce or delay dementia onset.	1. Retrospective cohort using administrative health data, no clinical cognitive tests done. 2. Possible under coding or misclassification bias. 3. The study included only definite dementia cases, so early cognitive decline may have been missed, potentially underestimating true dementia incidence.
13	Okamoto et al. (2015) [35] Japan	Community residents	Study design: Prospective Observational. Sample size: *n* = 3696 (gender not mentioned) Age group: Over 65 years	5 years	**CFs**: Age, Gender, education level, smoking habit, drinking frequency, history of taking oral corticosteroids or cytostatic drug **CMs**: Cancer, myocardial infarction, cerebrovascular disease, diabetes mellitus, hypertension, or dyslipidemia, anemia	1. Tooth loss predicts the development of Mild memory impairment in older adults. 2. Genetic risk factors related to both periodontal disease and mild memory impairment (MMI)	1. Results are biased by the exclusion of subjects who did not return for the follow-up examination. 2. MMSE classification may be inaccurate due to influence of age and education. 3. CPI index may not fully capture cumulative periodontal burden; radiographs would have been more suitable. 4. Causes of tooth loss (caries, trauma) not assessed, possibly overestimating PD-MMI relationship. 5. Timing of tooth loss not evaluated, making temporal relationship with MMI unclear. 6. APOE genotyping not performed, despite its known influence on CI risk.

### Sample size, age group and follow-up time

Sample sizes ranged widely, from 40 participants [27] in a German pilot study to over half a million participants in a Korean cohort [26]. The majority of studies included both male and female participants, though some focused only on one gender (males only) [25]. Sex distribution was not reported in several studies, and only a small minority provided sex-stratified effect estimates [28, 30–32, 35]. (Table 3)

The majority of studies focused on individuals aged 60 years and above. However, one long-term cohort enrolled adults from age 28; we retained it because follow-up captured cognitive outcomes in later life, but we interpret its findings cautiously regarding applicability to older adults [25]. Follow-up durations varied, with short-term (3–5 years) in some Japanese studies to long-term follow-ups exceeding 14 years in a large cohort study [26], allowing for examination of long-term associations between OH and cognitive decline.

### Descriptive characteristics based on research questions

#### Primary Research Q1: Association between PD, tooth loss, and cognitive decline among older adults

The majority of large-scale cohort studies [24, 26, 28, 30], along with a cross-sectional investigation [32], reported positive associations between severe PD, tooth loss, and increased risks of dementia, AD, or mild CI. Two studies [24, 34] reported lower hazard ratios for dementia among individuals who received periodontal treatment or preventive dental care. Increased vulnerability to PD-related cognitive decline was observed among women and individuals with comorbid conditions such as diabetes, hypertension, and hyperlipidemia [24, 25]. Several studies also emphasized the role of edentulism and reduced occlusal support, with findings indicating that impaired occlusal function may exert a stronger influence on cognitive decline than PD severity alone [31, 33].

In contrast, a subset of studies reported inconsistent or null findings. After adjustment for confounders, some investigations found no significant associations between PD parameters and CI [30, 33]. Other studies observed no predictive value of baseline PD status or tooth count for long-term cognitive decline, or reported that although PD was prevalent among individuals with dementia, periodontal status did not differ significantly across dementia subtypes [27, 31]. Ecological analyses indicated a statistical correlation between higher AD mortality and populations with greater prevalence of PD and subjective cognitive decline in populations with greater prevalence of PD and edentulism [32]. Overall, several studies reported associations between PD and tooth loss together contribute to cognitive decline among older adults [24, 26, 28, 32, 34] (Table 3).

#### Sub-research questions

##### Q1. Diagnostic approaches have been used to assess PD, tooth loss, and cognitive outcomes

All studies investigated the role of PD and tooth loss in relation to cognitive outcomes, using diverse diagnostic approaches.

PD was assessed using a range of clinical measures, including probing pocket depth, clinical attachment loss, bleeding on probing, plaque and gingival indices, radiographic bone loss, and periodontal inflamed surface area [27, 30]. In contrast, several studies relied on administrative or insurance databases, identifying PD based on ICD codes or dental treatment histories [24, 25, 34]. Some national health screening systems incorporated periodontal signs and calculus severity [26] (Table 4).

**Table 4:** Diagnostic approaches used to assess periodontitis, tooth loss, and cognitive outcomes (Q1)

Study (Author, Year, Country)	PD Diagnostic Approach	Clinical vs. Administrative PD Assessment	Tooth Loss Measure	Cognitive Assessment Method	Dementia Identification Method (if applicable)
Fadzli et al., 2024 [24] (Brunei)	Medical record diagnosis (acute/chronic gingivitis & periodontitis)	Administrative database (Bru-HIMS records)	Tooth extraction history	Not specified (clinical AD diagnosis recorded)	Alzheimer’s disease documented in Bru-HIMS records
Kaye et al., 2010 [25] (USA)	Alveolar bone loss progression; probing pocket depth progression	Clinical examination	Extraction history in treatment records	MMSE and Spatial Copying Task	Not based on ICD; cognitive testing used
Kim et al., 2020 [26] (Korea)	Dentist-assessed periodontal signs (gingival inflammation, calculus severity, tooth loss severity)	National Health Insurance screening system	Number of remaining and missing teeth	Diagnosis based on NINCDS-ADRDA guidelines	Specialist-diagnosed AD/VaD via insurance database
Laugisch et al., 2021 [27] (Germany)	Probing pocket depth (PPD), Clinical attachment loss (CAL), Bleeding on probing (BOP), Radiographic bone loss, Full-mouth plaque score, Periodontal Inflamed Surface Area.	Clinical full-mouth examination	Mean missing teeth; DMFT index	CERAD battery, MMSE, MRI patterns	NIA-AA 2011 diagnostic guidelines
Yamaguchi et al., 2023 [28] (Japan)	Probing pocket depth (PPD)	Clinical examination	Number of teeth present	MMSE	Not ICD-based; cognitive testing used
Luo et al., 2023 [29] (USA)	PPD and CAL (categorized PD severity)	Clinical examination	Significant tooth loss (≥ 8 missing vs. < 7)	MCI defined by NIA-AA criteria	Clinical MCI diagnostic criteria applied
Nilsson et al., 2018 [30](Sweden)	PPD and radiographic alveolar bone level (panoramic radiographs)	Clinical + radiographic examination	Number of teeth counted clinically	MMSE and Clock Test	No ICD; cognitive testing used
Naorungroj et al., 2015 [31] (USA)	PPD and bleeding on probing (BOP)	Clinical examination	Number of natural teeth; edentulism status	Three neuropsychological tests at both time points: a) Delayed Word Recall (DWR)- verbal memory, b) Digit Symbol Substitution (DSS)- psychomotor speed/attention, c) Word Fluency (WF)- executive/ language.	No clinical dementia diagnosis; neuropsychological testing
Scherer et al., 2020 [32] (USA)	State-level PD prevalence (CAL & probing depth)	Ecological epidemiological databases	Number of teeth lost (Self-reported survey) a) % of adults ≥ 65 years edentulous (all teeth lost). b) % of adults ≥ 65 years with ≥ 6 teeth lost.	State-level SCD and AD prevalence data	Administrative epidemiological records (not individual-level)
Hatta et al., 2018 [33] (Japan)	Mean periodontal pocket depth (PPD)	Clinical examination	Number of remaining teeth; posterior occlusal support	MoCA-J	Cognitive decline defined as ≥ 3-point MoCA-J decrease
Kamer et al., 2020 [14] (Denmark)	Modified Community Periodontal Index (MCPI)	Clinical examination	Number of missing teeth	Digit Symbol Test; Block Design Test	No clinical dementia diagnosis; cognitive performance tests
Lee et al., 2017 [34] (Taiwan)	ICD-9-CM periodontal disease codes (gingivitis, gingival recession, acute or chronic periodontitis).	Administrative insurance database	Tooth extraction as indicator of advanced PD	ICD-9-CM codes 290.X, 331.0	Dementia identified via insurance ICD codes
Okamoto et al., 2015 [35] (Japan)	Community Periodontal Index (CPI code)	Clinical examination (WHO probe)	Number of remaining teeth	MMSE; Word Recall; GDS short version	Mild memory impairment classification based on cognitive testing

Tooth loss was most frequently measured by the number of remaining or missing teeth; however, alternative indicators were also used, including edentulism, posterior occlusal support, significant tooth loss (e.g., ≥ 8 missing teeth or fewer than 10 remaining teeth), and extraction history [28, 29, 32–35]. Only one study applied the DMFT index to quantify tooth loss [27] (Table 3).

Cognitive impairment was evaluated using standardized neuropsychological assessments such as the Mini-Mental State Examination (MMSE) [25, 28, 30, 35] Montreal Cognitive Assessment (MoCA-J) [33], Clock Test [30], comprehensive neuropsychological test batteries [31] and CERAD battery [27]. In several studies, dementia and AD diagnoses were identified through clinical criteria based on established guidelines or through administrative data using ICD codes and medication records [24, 25, 34].

##### Q2: Extent of contribution to dementia and AD

Evidence from retrospective and prospective studies indicated that both PD and tooth loss contribute substantially to the risk of dementia and AD. Retrospective cohort studies (Taiwan and Brunei) reported significantly higher hazard ratios for dementia among individuals with PD and untreated dental conditions [24, 34]. A prospective observational study (Japan) demonstrated that tooth loss predicts the development of mild memory impairment in older adults [35]. Long-term follow-up data from large national cohorts (Korea) further showed that severe PD in combination with tooth loss significantly increased AD risk over periods extending up to 14 years [26]. Additional prospective studies (Japan) consistently confirmed that advanced tooth loss and PD were associated with subsequent cognitive impairment [28, 35].

##### Q3: Role of confounding factors and comorbid conditions

Most of the studies adjusted for age, sex, education, and lifestyle factors (e.g., smoking, alcohol, physical activity). Commonly reported comorbidities included diabetes mellitus, hypertension, cardiovascular disease, hyperlipidemia, and stroke. Several studies highlighted these as potential mediators or effect modifiers in the PD, tooth loss and CI relationship.

##### Q4: Gaps and limitations in the literature

Several gaps and limitations emerged across the included studies:

1. *Study design*: Cross-sectional dominance (4 of 13 studies) prevents causal inference.
2. *Diagnostic variability*: Heterogeneity in study designs, sample size, follow-up duration, diagnostic methods of PD, tooth loss, and CI (ranging from clinical examination to administrative coding and self-reported data) made comparisons difficult and contributed to inconsistent findings (Table 4).
3. *Confounding factors*: Adjustment for comorbidities such as diabetes, hypertension, and lifestyle factors were inconsistent. Also, inadequate adjustment for socioeconomic factors, education, lifestyle, and genetics (few studies adjusted for APOE ε4).
4. *Generalizability*: Population bias (male-only, younger dementia cohorts, or regionally restricted samples) limits generalizability.
5. *Data limitations*: Data source limitation reliance on administrative codes or self-reported oral health data risk misclassification.
6. *Biological mechanisms* (e.g., systemic inflammation/ inflammatory pathways, microbial spread/ oral microbiota translocation) were not directly measured in most studies (Table 3).

### Risk of bias assessment

The risk of bias assessment of the included cohort and cross-sectional studies revealed mixed methodological quality. Most cohort studies (Table 5) demonstrated strengths in clearly defining exposures, ensuring valid and reliable outcome measurement, and employing appropriate statistical analyses. However, several studies had notable limitations. For example, some failed to identify or adequately adjust for confounding factors [24, 27, 32] (Table 6), while others provided limited or unclear information regarding validity of exposure measurement [24, 33, 34]. Issues with follow-up were also evident, with incomplete reporting or lack of strategies to address attrition in a number of studies [24, 25, 33, 34] (Tables 5 and 6). Although the majority of studies applied appropriate statistical approaches, inconsistencies in exposure definitions, outcome ascertainment, and handling of potential biases reduced the overall comparability of findings across studies.

**Table 5:** Major components of the tools assessing cohort studies

Response Options									
Major Components	Fadzli et al. (01)	Kaye et al. (02)	Kim et al. (03)	Yamaguchi et al. (05)	Luo et al. (06)	Naorungroj et al. (08)	Hatta et al. (10)	Lee et al. (12)	Okamoto et al. (13)
1. Were the two groups similar and recruited from the same population?	No	Yes	Yes	Not applicable	Yes	Not applicable	Yes	Not Applicable	Yes
2. Were the exposures measured similarly to assign people to both exposed and unexposed groups?	Not applicable	Yes	Yes	Yes	Yes	Yes	Yes	Yes	Yes
3. Was the exposure measured in a valid and reliable way?	Unclear	Yes	Yes	Yes	Yes	Yes	Unclear	No	Yes
4. Were confounding factors identified?	No	Yes	Yes	Yes	Yes	Yes	Yes	Yes	Yes
5. Were strategies to deal with confounding factors stated?	No	Yes	Yes	Yes	Yes	Yes	Yes	Yes	Yes
6. Were the groups/participants free of the outcome at the start of the study (or at the moment of exposure)?	Yes	Yes	Yes	Yes	Unclear	Yes	Unclear	Yes	Yes
7. Were the outcomes measured in a valid and reliable way?	Yes	Yes	Yes	Yes	Yes	Yes	Yes	Yes	Unclear
8. Was the follow up time reported and sufficient to be long enough for outcomes to occur?	Yes	Yes	Yes	Yes	Yes	Yes	Yes	Yes	Yes
9. Was follow up complete, and if not, were the reasons to loss to follow up described and explored?	No	No	Yes	Yes	Yes	No	No	No	No
10. Were strategies to address incomplete follow up utilized?	Not applicable	No	Yes	Yes	Yes	No	No	No	Unclear
11. Was appropriate statistical analysis used?	Yes	Yes	Yes	Yes	Yes	Yes	Unclear	Yes	Yes
Comments	Included	Included	Included	Included	Included	Included	Included	Included	Included

**Table 6:** Major components of the tools assessing cross-sectional studies

Major Components	Laugisch et al. (04)	Nilsson et al. (07)	Scherer et al. (09)	Kamer et al. (11)
1. Were the criteria for inclusion in the sample clearly defined?	Yes	Yes	Unclear	No
2. Were the study subjects and the setting described in detail?	Yes	Yes	Yes	Yes
3. Was the exposure measured in a valid and reliable way?	Unclear	Unclear	Yes	No
4. Were objective, standard criteria used for measurement of the condition?	Yes	Yes	Yes	Yes
5. Were confounding factors identified?	No	Yes	No	Yes
6. Were strategies to deal with confounding factors stated?	No	Yes	No	Yes
7. Were the outcomes measured in a valid and reliable way?	Yes	Yes	Yes	No
8. Was appropriate statistical analysis used?	Yes	Yes	Yes	Unclear
Comments	Included	Included	Included	Included

## Discussion

While prior reviews have examined periodontal disease or tooth loss independently, this review specifically synthesizes studies that assessed both exposures concurrently within the same populations, enabling evaluation of cumulative oral disease burden in relation to cognitive outcomes. This approach allows for a more integrated assessment of cumulative oral disease burden in relation to cognitive outcomes, a perspective that is increasingly emphasized in the context of healthy ageing research [36]. The findings suggest that PD and tooth loss are consistently associated with an increased risk of CI and dementia, although the strength of associations varied depending on study design, diagnostic criteria, follow-up duration, and population characteristics [24, 26, 28]. In addition, although inconsistent findings because of causality could not be established due to study design (cross-sectional study) [30, 32], also demonstrated that history of tooth loss and PD may be important for cognitive function in older adults.

The publication trend revealed a substantial increase in related literature, indicating growing recognition of the topic among researchers. The following section presents the bibliographic analysis of the included studies. The authors’ country affiliations were extracted to assess the geographic distribution of the included studies. Research was conducted across diverse regions, including Asia (Japan, Korea, Taiwan, Brunei), Europe (Germany, Sweden, Denmark), and North America (USA), indicating broad international representation.

### Age and gender as modifiers

Age and gender emerged as important modifiers of these associations. Older age amplified the link, likely reflecting cumulative pathogen exposure and prolonged duration of tooth loss. However, several studies included relatively young to middle-aged populations (28–55 years) despite framing their focus on older adults [25–27, 29, 31, 32, 34]. This discrepancy undermines representativeness and limits generalizability to the older adults, who are at the highest risk for both oral disease and dementia.

Gender differences remain underexplored, sex distribution was not reported in several studies, and only a small minority provided sex-stratified effect estimates [28, 30–32, 35]. Only one study reported that women may be more vulnerable to PD-related dementia [24], while most failed to analyze sex as a biological variable. Broader epidemiological data show that women are disproportionately affected by AD [37, 38] and may experience more rapid periodontal destruction, potentially due to hormonal influences on both periodontal status and neurodegeneration [39]. Ignoring gender-sensitive analyses risks oversimplification and hinders development of tailored interventions. Sex distribution was not reported in several studies, and only a small minority provided sex-stratified effect estimates.

### Methodological and diagnostic heterogeneity

A key limitation across studies was diagnostic heterogeneity. Periodontitis was variably assessed using probing depth, clinical attachment loss, or self-report, while tooth loss was measured inconsistently through clinical exams, medical records, or self-reports. Similarly, cognitive outcomes ranged from standardized tools (MMSE) to clinical diagnoses of dementia. Such inconsistencies introduce misclassification bias and complicate cross-study comparison [40].

Follow-up duration also varied widely, from cross-sectional designs to longitudinal cohorts exceeding 10 years. Longer follow-ups strengthened evidence for temporality and dose-response relationships, whereas short-term and cross-sectional studies risked reverse causation-where cognitive decline itself contributes to poor OH [31]. This underscores the need for prospective designs with repeated, standardized assessments.

An additional source of heterogeneity relates to asymmetric exposure measurement. Although this SR included studies assessing both PD and tooth loss, some investigations emphasized one exposure more strongly than the other, or used proxy indicators such as tooth extraction history to represent advanced periodontal disease. Such variability in exposure operationalization may introduce misclassification and contribute to inconsistencies in effect estimates across studies [41].

### Comorbidities and confounding factors

Findings suggest that comorbidities such as diabetes, hypertension, and hyperlipidemia influence the oral-cognitive pathway to varying degrees. Periodontal inflammation exerts systemic metabolic effects relevant to both peripheral and brain health [42]. Epidemiological evidence also supports shared risk profiles between vascular disease, dementia, and periodontal breakdown [37, 38].

In line with this, previous studies reported a high prevalence of CI among older persons with chronic illnesses, particularly those with lower education levels, high blood pressure, and diabetes, highlighting the compounding effects of systemic health conditions on cognitive decline [43, 44].

Confounding remains another critical challenge. While some studies adjusted for age, education, smoking, diabetes, and cardiovascular disease, others provided only minimal adjustment. Socioeconomic status and access to dental care - powerful determinants of OH - were rarely considered, despite their potential to bias results [38]. Neglecting these structural factors not only risks overestimating associations but also obscures the pathways linking oral and cognitive health.

### Implications for future research, clinical practice, and public health policy

The findings of this SR have important implications for research, clinical practice, and public health policy. A multidisciplinary approach to OH care is particularly important in older populations, where OH status may influence treatment outcomes, recovery, and overall functional capacity, as highlighted by Briguglio and Wainwright [45]. At the health system level, incorporating routine OH assessments into primary care offers opportunities for early risk identification and preventive intervention. In line with this, the FDI World Dental Federation [46] advocates for the systematic inclusion of OH within national ageing and public health strategies, including training non-dental healthcare professionals in OH screening and integrating dental care into routine geriatric services.

Addressing broader social determinants of OH such as income, education, and access to care, is equally critical, as poor OH continues to disproportionately affect vulnerable and ageing populations. Patel and Gallagher [47] emphasize that an integrated, life-course approach is needed to respond effectively to the OH needs of ageing societies. Supporting this perspective, a recent review [48] reported strong long-term associations between OH particularly the retention of a greater number of natural teeth and trajectories of healthy ageing, with associations remaining robust after adjustment for multiple confounders. These findings reinforce the need to embed OH within broader healthy ageing and public health policies, recognizing that maintaining natural dentition may support physical health, cognitive function, nutrition, social participation, and quality of life in later life.

### Risk of bias and quality of evidence

The overall risk of bias across included studies was high, driven by selection bias, reliance on self-reported oral health measures, heterogeneous cognitive assessments, incomplete follow-up, and limited strategies to address attrition. Potential exposure misclassification and incomplete confounder control (e.g. not adjusting for education or comorbidities) could bias associations toward the null or exaggerate them. This underscores the need for cautious interpretation of the observed associations. In addition, the predominance of studies from high-income countries restricts generalizability to low- and middle-income settings, where the burden of both oral disease and dementia is rapidly increasing. Strengthening the evidence base will require improved methodological rigour, including standardized diagnostic criteria, comprehensive confounder adjustment, representative sampling, and incorporation of biological markers such as inflammatory mediators or periodontal pathogens (e.g., *Porphyromonas gingivalis*) to better elucidate underlying mechanisms [24].

### Limitations of the study

This SR has several limitations. First, heterogeneity in study design, an additional source of heterogeneity arises from asymmetric exposure assessment across studies. Some investigations emphasized either periodontitis or tooth loss, or relied on proxy indicators such as tooth extraction history to reflect periodontal burden. While these measures capture cumulative oral disease exposure, they may differ in sensitivity and specificity, potentially contributing to variability in observed associations. Diagnostic criteria, and outcome measures limited direct comparisons and meta-analysis. Second, as all available studies were included due to the absence of prior SRs on this combined topic, the findings must be interpreted cautiously. Third, most included studies were observational, preventing causal inference. Finally, language restrictions and database selection may have excluded relevant studies.

Despite these limitations, the SR provides novel insights by addressing PD and tooth loss together, strengthening the case for considering oral frailty in cognitive health research. At last the age threshold for defining ‘older adults’ varied across studies, which may affect comparability of the findings.

## Conclusions

This SR synthesized observational evidence on associations between PD and tooth loss and CI outcomes in older adults. Across included studies, poorer periodontal status and greater tooth loss were generally associated with worse cognitive outcomes; however, heterogeneity in definitions, residual confounding and potential reverse causation limit causal inference. Future longitudinal studies using standardized periodontal assessment and consistent cognitive outcome ascertainment are needed to clarify temporality and strengthen inference.

Well-designed longitudinal studies using standardized oral and cognitive assessments and comprehensive control of biological, behavioral, and social confounders are needed to clarify temporality. Clinically and from a public health perspective, integrating oral health into strategies for healthy cognitive ageing may offer preventive potential, particularly through routine assessment, periodontal care, and timely management of tooth loss within equitable, patient-centered care models.

## Supplementary Information

Supplementary Material 1.

Supplementary Material 2.

## Data Availability

Data will be available from the corresponding author upon reasonable request.

## Abbreviations

AD: Alzheimer’s disease
PD: Periodontitis
CI: Cognitive Impairment
OH: Oral Health
