# Supplementary material for: Association of periodontitis and tooth loss with cognitive decline in the older adults - a systematic review
Source: BMC Oral Health. 2026 Apr 11;26:811. doi: 10.1186/s12903-026-08310-w (PMC13156852; doi:10.1186/s12903-026-08310-w)
Supplement: Supplementary file 2 — Supplementary Material 2. [file 12903_2026_8310_MOESM2_ESM.docx]

| **Database** | **Query** | **Notes** | **Records retrieved** | **Filter** |
| --- | --- | --- | --- | --- |
| PubMed (via MEDLINE) | (  ("Periodontal Diseases"[Mesh]  OR "Periodontitis"[Mesh]  OR periodontitis[tiab]  OR paradontosis[tiab]  OR "periodontal disease"[tiab]  OR "periodontal inflammation"[tiab]  OR "periodontal infection"[tiab]  OR "gum disease"[tiab]  OR "gum inflammation"[tiab]  OR "gum infection"[tiab]  OR gingivitis[tiab]  OR "Tooth Loss"[Mesh]  OR "tooth loss"[tiab]  OR "missing teeth"[tiab]  OR edentulism[tiab]  OR edentulous[tiab]  OR "tooth extraction"[tiab]  OR "dental extraction"[tiab])  AND  (("Dementia"[Mesh]  OR "Alzheimer Disease"[Mesh]  OR "Cognitive Dysfunction"[Mesh]  OR "Cognition Disorders"[Mesh]  OR dementia[tiab]  OR "cognitive decline"[tiab]  OR "cognitive impairment"[tiab]  OR "mild cognitive impairment"[tiab]  OR "major neurocognitive disorder"[tiab]  OR "mild neurocognitive disorder"[tiab]  OR "Alzheimer disease"[tiab]  OR "Alzheimer's disease"[tiab]  OR "neurocognitive disorder"[tiab]))  AND  (("Aged"[Mesh]  OR "Aging"[Mesh]  OR "Healthy Aging"[Mesh]  OR older[tiab]  OR "older adult*"[tiab]  OR "older person*"[tiab]  OR "older population"[tiab]  OR "older people"[tiab]  OR elderly[tiab]  OR elders[tiab]  OR "elder population"[tiab]  OR "elder people"[tiab]  OR "aging population"[tiab]  OR geriatric[tiab]  OR senior[tiab]  OR "senior citizen*"[tiab]  OR "healthy aging"[tiab])) | Used **MeSH + keywords**; balanced specificity and sensitivity. | 419 | *Publication date: March 2010 to March 2025  *Document type: Article  *Language: English |
